# Can altered magnetic field affect the foraging behaviour of ants?

**DOI:** 10.1371/journal.pone.0225507

**Published:** 2019-11-25

**Authors:** Márlon César Pereira, Ingrid de Carvalho Guimarães, Daniel Acosta-Avalos, William Fernando Antonialli Junior

**Affiliations:** 1 Programa de Pós-graduação em Entomologia e Conservação da Biodiversidade, Universidade Federal da Grande Dourados, Dourados, Mato Grosso do Sul, Brazil; 2 Laboratório de Ecologia Comportamental, Center of Studies on Natural Resources, Universidade Estadual de Mato Grosso do Sul, Dourados, Mato Grosso do Sul, Brazil; 3 Programa de Pós-graduação em Recursos Naturais, Universidade Estadual de Mato Grosso do Sul, Dourados, Mato Grosso do Sul, Brazil; 4 Centro Brasileiro de Pesquisas Físicas, Rio de Janeiro, Rio de Janeiro, Brazil; University of Sheffield, UNITED KINGDOM

## Abstract

Social insects such as ants can use geomagnetic field information in orientation and navigation tasks. However, few studies have assessed the effect of magnetic fields on aspects such as orientation and decision making during foraging of ants. Therefore, the present study aims to test the hypothesis that foragers of different species of ants with different foraging strategies when under effect of applied magnetic field change the patterns of search for resources and recruitment of ants. We used two species with solitary foraging strategy, *Ectatomma brunneum* and *Neoponera inversa*, and another with mass recruitment, *Pheidole* sp. The experiments were performed in field and laboratory conditions. We used some parameters for comparison such as speed, distance and time during foraging in the field and laboratory experiments, under normal and applied magnetic field with the coils on and off. We also performed SQUID magnetometry analysis for all species. The results demonstrate that changes in normal values of magnetic field affect workers behaviour of the three species. Thus, we can conclude that ants under the effect of applied magnetic fields can suffer significant changes in their foraging activities decreasing the flow of workers, increasing the travelled distance from the nest to the resource and back to the nest, in addition to time and distance to fetch the resource and decision-making, in both types of species, those which have mass recruitment, or forage individually, and that the three species are magnetosensitive, being affected by changes of low intensity in the local magnetic field.

## Introduction

The geomagnetic field is an abiotic component, which interacts permanently with living beings and has been present since the emergence of life on Earth [1]. Many animals are sensitive to magnetic fields [2–4]. In the case of marine environments, animals can use electroreceptor organs to detect orientation cues from the geomagnetic field [3,5]. Animals can use the direction of the geomagnetic field vector as a compass during homing or migratory activities [3,4]. It is known that animals can use two different magnetoreception mechanisms: the detection of the geomagnetic field can be based on sensitive light-dependent chemical reactions, occurring in chromophores as the cryptochrome [3,6,7] or on physical processes involving magnetic nanoparticles (the ferromagnetic hypothesis) [2,6,8,9].

Several experiments have shown that crustaceans [10], amphibians [11], fish [12], reptiles [13], birds [14,15], mammals [16], insects [17,18] and, in particular, social insects such as bees, ants and wasps, can use the geomagnetic field information in orientation and navigation tasks [18,19]. The efficient orientation mechanisms of eusocial insects make them a particularly interesting group for studies on magnetic orientation, due to the large number of individuals in their colonies, which allows many experimental observations in short periods of time [18]. The organization of their colonies relies on tasks of a considerable degree of complexity, with communication and foraging performed utilising different sensory abilities based on: visual, acoustic, tactile, magnetic and/or chemical information [20]. Some species may forage solitarily without any kind of recruitment, while others optimize resource exploitation by recruiting nestmates through chemical trails [21,22].

Ants, in particular, are social insects that represent an evolutionary branch that emerged 50 million years ago [21], and were the first social predator group to explore the soil and vegetation [21]. The search strategies for resources in colonies of these insects can vary. Some species may present solitary foraging behaviour, in which workers look for resources alone without cooperation from their nestmates. That behaviour is found in less derived species. On the other hand, some more derived species show foraging behaviour in groups by pheromonal trail marking, in which the first worker that locates the resource returns to the nest marking its trail for the next to follow and find the resource [21,22].

Many ant species produce large branching patterns of trails around their nests [23]. These patterns, which represents the space exploration and foraging activities coordination throughout the colony, are among the most important examples of transport networks built by animals [21,24] and channelize the daily movements of hundreds or thousands of ants. Models [25,26] and empirical observations [27–30] have shown that ant trail networks provide efficient solutions to transport and search for food.

A quick and efficient exploration of resources is essential for the maintenance of an ant colony in environments under modification and with high prey variety. The colonies respond with high adaptive allocation of foragers to adjust to the availability of variable resources. Particularly in large colonies, as in army ants species [31] and leaf-cutting ants [32], the number of foragers can easily exceed thousands of individuals when resources are highly available. The spatial distribution of these large numbers of foragers can be particularly challenging for ant species that forage along permanent trail systems that are cleaned and maintained by ants. These trails have been described in Myrmicinae, Dolichoderinae and Formicinae [21].

Magnetoreception has been observed in several animals, from insects to mammals [33,34]. In the case of insects the studies have been concentrated on some bees, especially honeybees, one wasp species, migratory butterflies and ants [18,35]. The few studies regarding magnetoreception in ants have focused on the relation between the magnetic field direction and spatial orientation. Some studies have shown that ants are sensitive to magnetic fields: *Acromyrmex octospinosus* Forel 1899 avoid areas with high magnetic field intensity [36], *Solenopsis invicta* Buren 1972 change the time for trail formation [37] and *Formica pratensis* Retzius 1783 relates areas in a specific geomagnetic field direction to food sources [38]. On the other hand, other studies have shown that several species of ants are able to use the geomagnetic field as an orientation cue: *Oecophylla smaragdina* Fabricius 1775 [39], *Neoponera* (= *Pachycondyla*) *marginata* Roger 1861 [40], *Atta colombica* Guérin-Méneville 1844 [41], *Solenopsis sp*. [42] and *Cataglyphis nodus* Brullé 1833 [43,44]. There is no doubt that ants are able to orient following the geomagnetic field, but until now none of those studies asked whether ants trajectories during foraging activities are sensitive to changes in the ambient magnetic field. As mentioned above, ant's foraging activities are important for nest maintenance. Therefore, considering that technological developments led to the emergence of devices that can cause change in natural values of magnetic field of the planet [45], it is possible that this phenomenon may change the natural behaviour of ant colonies.

As far as we know, no other study has shown the effect of magnetic fields on the foraging behaviour, the only one to do that being the study of the time for trail formation in *S*. *invicta* [37]. Therefore, the present study aims to test the hypothesis that foragers of different species of ants with different foraging strategies when under effect of applied magnetic field change the patterns of search for resources and recruitment of ants.

## Materials and methods

The collection of the material reported in this study complied with the current applicable federal law of Brazil (permanent license for collection of Zoological Material No. 17487–1 MMA/ IBAMA/ SISBIO to WFAJ).

To test the effect of applied magnetic fields on the foraging activity of ants, a digital power supply connected to Helmholtz coils was used as described by Pereira-Bomfim et al. [19]. The coils had 30 cm diameter with a space of 15 cm between them and were built with 58 spirals of Cu wire 14 AWG, capable of generating an static magnetic field of 60 μT. In addition, Pereira-Bomfim et al. [19] found that this value caused changes in foraging behaviour in social wasps. The effect of the artificial magnetic field on foraging behaviour was tested in two species with solitary foraging strategy, *Ectatomma brunneum* Smith 1848 and *Neoponera inversa* Smith 1848, and another with mass recruitment, *Pheidole* sp. The experiments were performed in field conditions with *Pheidole* sp. ants and in laboratory conditions with *E*. *brunneum* and *N*. *inversa* ants.

### Field experiments

We selected 10 colonies of *Pheidole* sp. nested in the surroundings of the campus of the Universidade Estadual de Mato Grosso do Sul (Dourados, MS, Brazil; 22° 11’55.44” S, 54°55’48.88” W), in an area of pasture, with predominance of grass and shrub species. The collection of the material reported in this study complied with the current applicable federal law of Brazil (permanent license for collection of Zoological Material No. 17487–1 MMA/ IBAMA/ SISBIO to WFAJ). In order to make sure that all colonies were from the same species, samples were sent to an expert for confirmation. The geomagnetic field at these geographic coordinates corresponds to a horizontal component of 19.8 μT, a vertical component of -10.6 μT and a total component of 22.5 μT. Therefore, the value of the applied magnetic field was about three times higher.

We placed a sheet of ethylene vinyl acetate (EVA) 50 x 25 cm, completely covered with a sheet of graph paper, always leading towards the north (N) at a distance of 5 cm from the nest entrance. At the farthest point from the nest entrance, we offered 25 g of attractive bait of honey and 25 g of pilchards as a resource.

**Fig 1:**
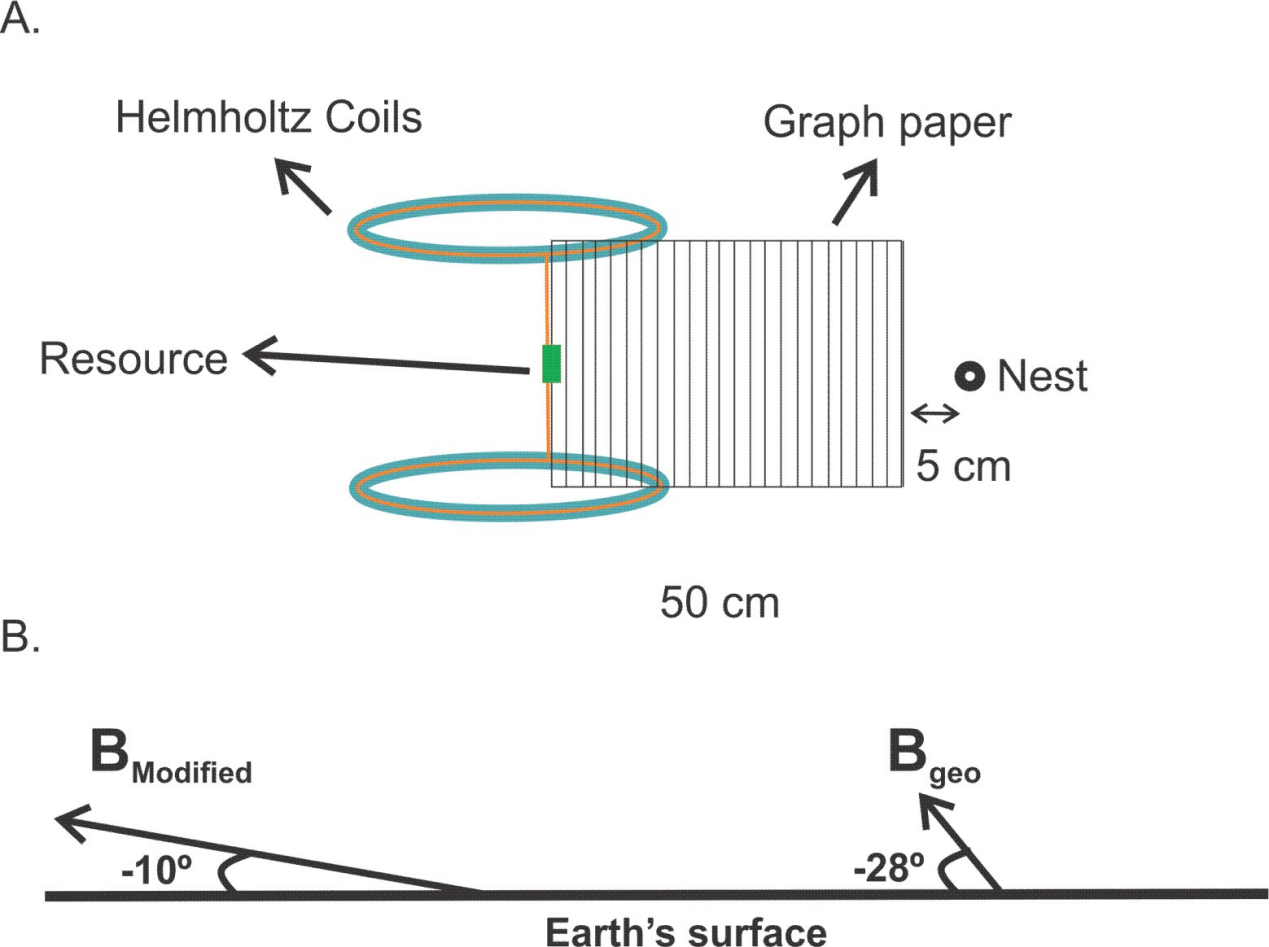
Diagram of the field experimental setup. (A) Diagram of the field experimental setup showing the position of Helmholtz coils, graph paper, nest entrance and resource offered for ants of the species *Pheidole* sp. (B) Diagram showing the normal geomagnetic field (B_GEO_) and the applied magnetic field (B_MODIFIED_) between the Helmholtz coils. B_MODIFIED_ is almost 3 times B_GEO_. The inclination angle is measured relative to the horizontal surface (Earth’s surface) and it changes from -23° to -10°. The inclination is negative because the magnetic field vector dip angle is up.

To assess the effect of the magnetic field on foraging behaviour of the species, the resource was allocated between the two Helmholtz coils, at 7.5 cm from each one of the coils (Fig 1). The magnetic field generated between the coils lasted 1 hour and during this period the following parameters were evaluated: 1) departure trajectory of the first worker, called W1 (DT1), 2) time that W1 took to get to the resource (T1), 3) time W1 remained in the resource, (TRR1), 4) trajectory of return to the nest of W1 (TR1), 5) time to return to the nest (TRN1), 6) speed during the departure trajectory (SD1), 7) speed during the return to the nest trajectory (SR1), 8) trajectory of the first worker recruited to follow the chemical trail, called W2 (DT2), 9) time this worker took to get to the resource (T2), 10) time W2 remained in the resource (TRR2), (11) trajectory of return (TR2), 12) time to return to the nest (TRN2), 13) speed during the departure trajectory (SD2) and 14) speed during the return to the nest trajectory (SR2).

A pin was placed every 5 seconds during the movement of each ant on the graph paper in order to outline its trajectory. In addition, the amount of workers arriving at the resource and returning to the nest was registered every minute in order to establish the foraging flow per hour (FF). Time parameters were measured in seconds and speed in cm/s. As a control experiment, we used the same methodology but with the power supply disconnected to maintain the geomagnetic field unaltered.

The trajectories obtained were analysed using the OVS Program following the foraging methodology according to Cammaerts et al. [46]. This software provides as trajectory parameters the distance travelled, the mean of the orientations along the curve (orientation) and the sinuosity of the curve, which corresponds to a measure of the deviation of the trajectory to that corresponding to a straight line.

### Laboratory experiment

We evaluated the foraging behaviour and decision-making of 10 foragers of each one of the six colonies of *N*. *inversa* collected in the Centro de Pesquisa da Lavoura Cacaueira in Ilhéus, BA, Brazil (14°45’40.36”S, 39°13’41.02” W), nested in a cocoa farming area; and four colonies of *E*. *brunneum* collected in the vicinity of the campus of the Universidade Estadual de Mato Grosso do Sul (UEMS) in Dourados, MS, Brazil. After collection, colonies were housed in artificial nests, built in plaster simulating the nesting patterns of the species in the environment, at the Laboratório de Ecologia Comportamental (LABECO/UEMS), kept at a temperature of 22 ± 2°C and photoperiod of 12:12 light/dark, fed with larvae of *Tenebrio molitor* Linnaeus 1758, molasses and water *ad libidum*.

**Fig 2:**
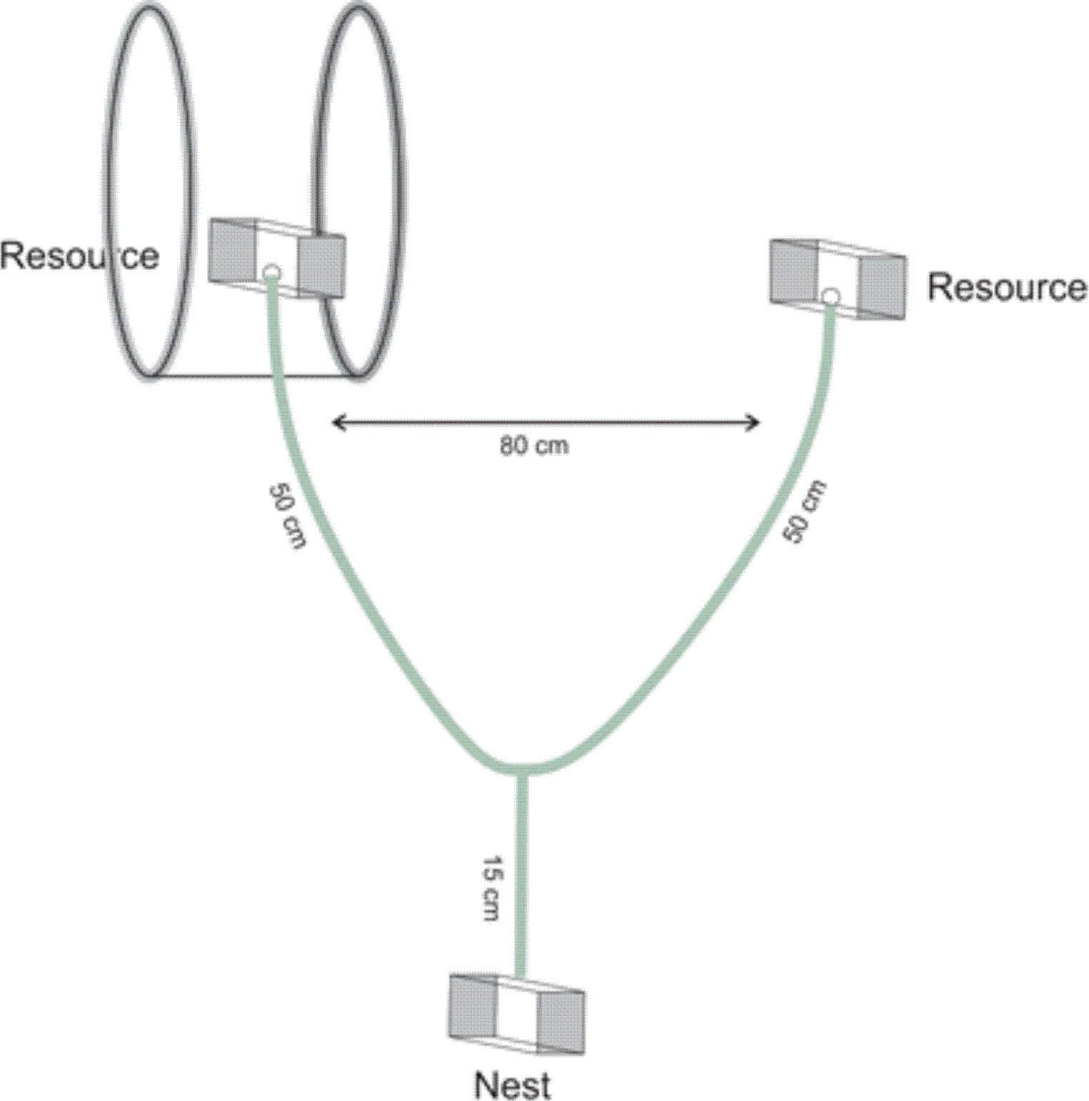
Diagram of the decision-making experimental setup using *Ectatomma brunneum* and *Neoponera inversa* ants.

Each artificial nest was coupled to a Y-maze of 2.5 cm diameter and 65 cm length (Fig 2), and a Gerbox® 250 ml plate was connected at each end. Thus, two options of trajectory were provided for foragers, in the extremities of which was offered the same type of attractive bait as resource, a mixture of 50 g of honey and 50 g of pilchards (Fig 2).

Soon after the placement of the resources, two ants at the same time were allowed to access the Y-maze through the opening of the nest entrance, using a piece of plastic in rectangular shape to control the passage of the ants. Two workers were allowed at the same time because we wanted to test whether the applied MF could change not only the decision-making of the ants, but also if coming into contact with the applied MF could change other aspects of their standard behaviour. After the entry of workers we assessed the following parameters 1) which end the ant has chosen, 2) time to get to the chosen end (TE), 3) mean speed of displacement inside the maze until reaching the resource (S), speed of return to the nest (SR) and 4) behaviour of foragers while moving through the maze, by *ad libitum* method [47]. We assessed the decision-making of 10 workers of each colony. After the end of the decision-making assessments of the workers, the maze was cleaned with alcohol so that possible odours released by these ants would not affect the decision of following ants.

To evaluate the effect of magnetic field on these parameters a pair of Helmholtz coils was placed at on one end of the Y-maze, with each coil at 7.5 cm from the resource provided, generating a magnetic field of 60 μT. These coils were 1 meter distant from the power supply to ensure that there was no interference with the experiment and the value of the magnetic field measured at the other end was approximately 20 μT, i.e. without modification of the natural magnetic field.

As a control, the experiment was also performed with the coils positioned in the same place but switched off, therefore with normal values of MF, so it was possible to define if the presence of the coils itself could change the behaviour and decision-making of the ants.

### SQUID magnetometry

Magnetometry was done to assess whether there are magnetic nanoparticles on the ants bodies. Individuals of *Neoponera inversa* (NI), *Ectatomma brunneum* (EB) and *Pheidole* sp. (PSP) were collected, and the heads, thoraxes and abdomens were separated, kept in the fridge and preserved in a solution of 70% alcohol. We used a SQUID magnetometer (MPMS Quantum Design) to search for the presence of magnetic material in these body parts. We defined the number of samples of workers from each species used for analyses according to their body size. Thus, 5 abdomens, thoraxes and heads of EB, 4 abdomens, thoraxes and heads of NI, and 20 abdomens, thoraxes and heads of PSP were dried and placed inside a gelatine capsule. This capsule was placed in a plastic straw that can be fixed on the magnetometer’s sample holder for magnetization measurements. Two types of magnetization measurements were done as a function of temperature: Zero Field Cooling (ZFC), where the sample is cooled from room temperature in the presence of a null magnetic field; and Field Cooling (FC), where the sample is cooled in the presence of a magnetic field, which was of 100 Oe (1 Oe = 100 μT) in our measurements. After cooling the sample, a magnetic field is applied and the magnetization is measured as the temperature increases. The range of temperature was between 10 K and 330 K and a magnetic field of 100 Oe was used. The ZFC and FC curves are used to identify the presence of magnetic nanoparticles in the size range of superparamagnetism (SPM) or single domain (SD). SD particles have a magnetic moment fixed to the particle in such a way that to change the magnetic moment direction the whole particle must change its direction. In the other way, SPM particles have a magnetic moment free to rotate inside the particle body and it is strongly influenced by thermal energy, making the magnetic moment change its direction randomly inside the nanoparticle. In SPM particles there is a temperature that limits the effect of the thermal energy: the blocking temperature T_B_. For temperatures higher than T_B_ the magnetic moment changes its direction randomly but the nanoparticle body maintains its direction the whole time, and the net magnetic moment of the nanoparticle is zero. For temperatures lower than T_B_ the magnetic moment becomes stable inside the nanoparticle (the magnetic moment is blocked) and now to change the magnetic moment direction is necessary to change also the nanoparticle orientation. This information is important to understand the possible ways that an animal can detect the geomagnetic field. ZFC and FC curves are also used to identify the presence of magnetite because of the Verwey transition at 120 K, identified for a sudden change in the magnetization in the region of the transition [48]. Hysteresis measurements (curves of magnetization M versus magnetic field B) at 300 K or 150 K were made on a magnetic field range between -10000 Oe and +10000 Oe. In the presence only of paramagnetic particles or SPM nanoparticles at T > T_B_ the M versus B curve is a single curve with S form, but in the presence SD nanoparticles or SPM nanoparticles at T < T_B_ the curve from +10000 Oe to -10000 Oe is not equal to the curve from -10000 Oe to +10000 Oe, showing what is known as hysteretic behaviour. Hysteresis curves can be used to identify in a better way the presence of SD nanoparticles, blocked SPM nanoparticles and pseudo-single domains (PSD) that are nanoparticle a little bit bigger than a SD. In the hysteresis curves we determined the saturation magnetization (M_S_), remanent magnetization (M_R_), coercive field (H_C_) and remanent coercive field (H_CR_). M_S_ is the result of the sum of all contributions to the magnetization (paramagnetic, SD and SPM) and M_R_ is composed solely by the ferromagnetic component (SD and blocked SPM nanoparticles). As heads, thoraxes and abdomens have different sizes and masses, we calculated M_S REL_ and M_R REL_ dividing the value of M_S,R_ by the total mass of the parts analysed. The values were used to compose the Day plot [49,50] (M_R_/M_S_ vs H_CR_/H_C_) which allows the separation of the type of magnetic particle in single-domain (SD), multidomain (MD), pseudo-single-domain (PSD) or mixture of SD + MD, and mixtures of SD + superparamagnetic (SPM).

### Statistical analyses

To assess the effect of the applied magnetic field on the activity of *Pheidole* sp. in the field, all the mean values of the parameters evaluated, with and without the coils being activated, were compared by a T test, after performing a normality test. The parameters obtained from the OVS software (distance, orientation and sinuosity) were compared in two ways: a paired T test was used to compare the parameters of departure and return trajectories, for each magnetic field condition (with and without) for all workers 1 (W1) and all workers 2 (W2); and an unpaired T test was used to compare the parameters of the departure trajectories with and without the applied magnetic field, and the same was done for the return trajectories, for all W1 and W2 ants. The *Instat* software was used in this case.

To assess the effect of the applied magnetic field on the behaviour and decision-making of foragers of *E*. *brunneum* and *N*. *inversa* under laboratory conditions, we performed a chi-square test, using the path chosen by the ants as variables (with or without magnetic field). A T test was performed to compare the mean speed of displacement between ants that have chosen each different path (with or without magnetic field).

## Results

**Fig 3:**
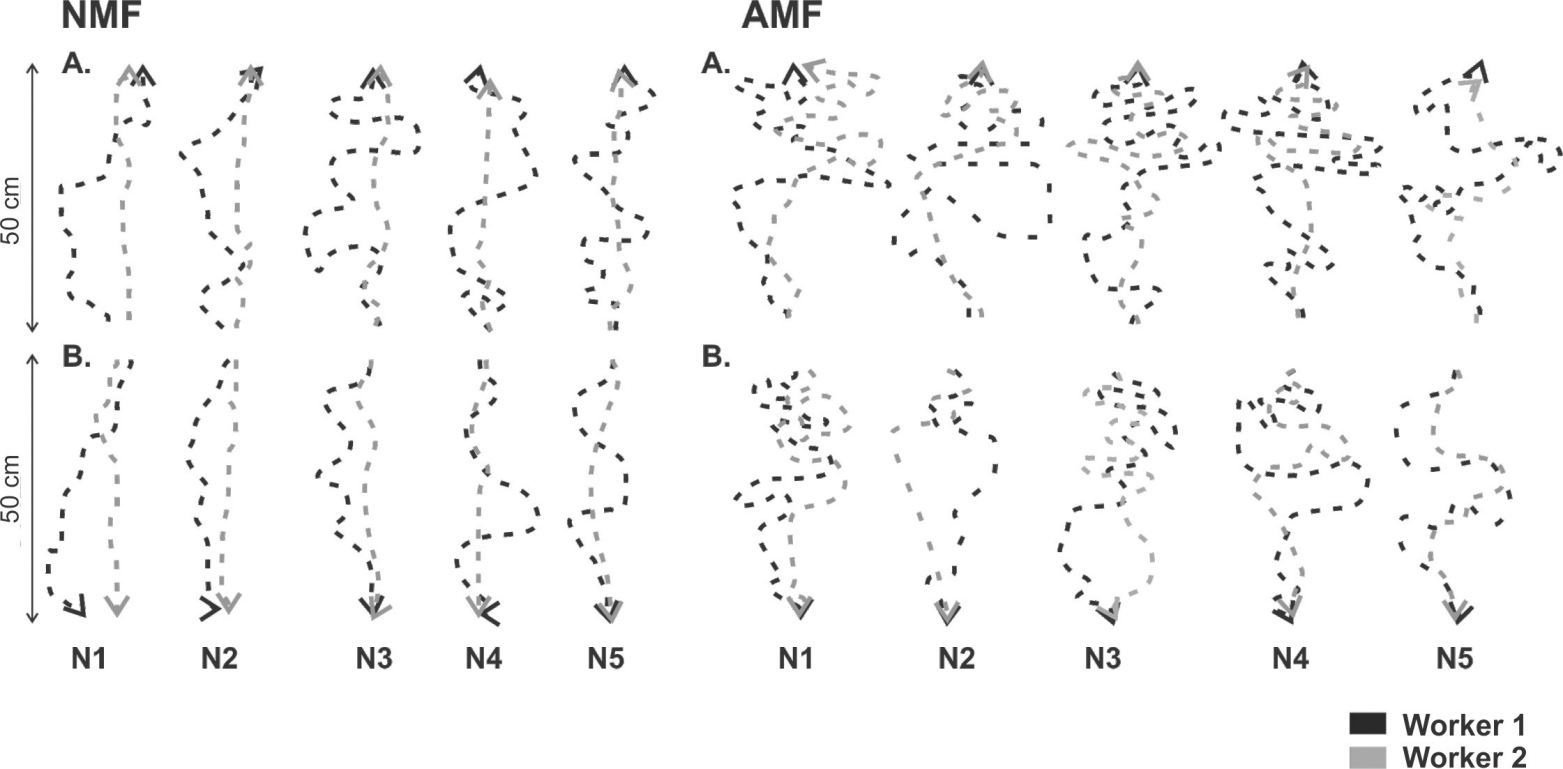
Trajectories of two *Pheidole* sp. workers foraging under normal (NMF) and applied (AMF) magnetic field. (A) Patterns of departure trajectories and (B) Patterns of return to the nest trajectories, from 5 different colonies. N1-5 = Nests 1 to 5.

We can observe by visual inspection of some trajectories of *Pheidole* sp. workers that there is a change between the condition with normal MF and applied MF (Fig 3 and S1–S10 Figs).

Indeed, there are significant differences between all mean values of all the parameters assessed, with only one exception, the W1 speed during the departure trajectory (SD1) (Table 1). The same happened with the parameters of the trajectories: distance, orientation and sinuosity (Table 2). All the parameters are statistically different (T test, p < 0.05) between the experimental conditions with normal magnetic field (NMF) and applied magnetic field (AMF), except the orientation for the return of W1. We can also observe that most of the parameters of the trajectories are statistically different (T test, p < 0.05) between departures and returns of W1 and W2 (Table 2). However, orientation and sinuosity with applied MF of W1, and distance with normal MF and sinuosity with applied MF of W2 are not statically significant (T test, p > 0.05) (Table 2).

**Table 1:** T test with the parameters analysed in field experiments with the applied Magnetic Field (MF).

Species	Normal MF	Applied MF	p (T test)
***Pheidole* sp. 60 Δ**			
DT1 (cm)	70.9±11.6	107.4±15.7	0.000001
TR1 (cm)	62.2±8.9	79.1±10.1	0.0009
T1 (s)	133.2±63.8	215.5±33.4	0.005
TRR1 (s)	149.9±87.3	33.0±11.5	0.004
TRN1 (s)	115.3±45.1	270.2±101.3	0.0004
SD1 (s)	0.73±0.5	0.47±0.1	0.19
SR1 (cm/s)	0.61±0.2	0.34±0.1	0.008
DT2 (cm)	51.7±1.5	70.1±14.5	0.0009
TR2 (cm)	52±0.4	66.9±11.9	0.0009
T2 (s)	31.7±6.6	157.9±91.8	0.001
TRR2 (s)	199.9±78.3	32.1±9.8	0.000007
TRN2 (s)	36.9±12	152.6±80.5	0.001
SD2 (cm/s)	1.71±0.4	0.66±0.5	0.0001
SR2 (cm/s)	1.6±0.6	0.73±0.8	0.02
FF (Δ/h)	1.51±0.5	0.53±0.3	0.0001

DT1 = departure trajectory of W1; TR1 = trajectory of return to the nest of W1; T1 = time that W1 took to get to the resource; TRR1 = time remained in the resource; TRN1 = time to return to the nest; SD1 = speed during the departure trajectory; SR1 = speed during the return to the nest trajectory; DT2 = trajectory of W2; TR2 = trajectory of return; T2 = time this worker took to get to the resource; TRR2 = time remained in the resource; TRN2 = time to return to the nest; SD2 = speed during the departure trajectory; SR2 = speed during the return to the nest trajectory; FF = foraging flow; Δ = individuals, s = seconds, cm = centimetres. W1 = First worker searching for resource; W2 = first worker following the chemical trail left by W1.Values expressed as Mean ± Standard Deviation.

**Table 2:** Parameters of the departure and return to the nest trajectories of workers of *Pheidole* sp. with normal MF and applied MF.

Parameters OVS	Departure NMF	Departure AMF	Return NMF	Return AMF
**W1**				
Orientation (°)	66.9±3.9^a,I^	73.9±3.4^A,II^	113.4±5.8^a,1^	108±6^A,1^
Distance (cm)	70.9±11.6^a,I^	107.4±15.7^A,II^	61.3±9.2^b,1^	79.1±10.2^B,2^
Sinuosity	12±3.2^a,I^	15±2.2^A,II^	8.7±2.8^b,1^	13.6±4.2^A,2^
**W2**				
Orientation (°)	56±1.9^c,III^	64.1±6.8^C,IV^	116.8±2.6^d,3^	111.5±4.1^D,4^
Distance (cm)	51.9±1.6^c,III^	70.1±14.5^C,IV^	52±0.5^c,3^	66.9±11.9^D,4^
Sinuosity	4.4±1.7^c,III^	11.6±2.9^C,IV^	2.8±1.2^d,3^	12.6±5.1^C,4^

NMF = Normal magnetic field; AMF = Applied magnetic field; W1 = First worker searching for resource; W2 = First worker following the chemical trail left by W1. Values expressed as Mean ± Standard Deviation. Letters represent the comparison between departure and return with NMF (small letters) and between departure and return with AMF (upper case letter): different letters mean significant difference (paired T test, p < 0.05). Numbers represent the comparison between departure with NMF and departure with AMF (Roman numerals) and between return with NMF and return with AMF (Latin numerals): different numbers mean significant differences (unpaired T test, p < 0.05).

There was a significant difference between the decision-making choices of workers of *E*. *brunneum* (χ^*2*^ = 34.8, p < 0.05) and *N*. *inversa* (χ^*2*^ = 53.9, p < 0.05) between the conditions with normal MF and with applied MF (Table 3). There was a significantly higher number of choices for the path leading to the resource that was not placed between the activated coils. No significant difference was found between the decision-making when the coils were off. There are significant differences between the mean speeds of displacement of ants inside the maze and mean time to get to the resource between the conditions, with normal MF and applied MF.

**Table 3:** T test with the parameters analysed in laboratory experiments with the applied magnetic field (MF).

Species	Normal MF	Applied MF	p (T test)
***E*. *brunneum* 40 Δ** **Control**			
TE (s)	98.2±17.5	93.9±12.9	0.38
S (cm/s)	0.68±0.1	0.70±0.1	0.59
SR (cm/s)	0.97±0.4	1.01±0.3	0.75
***E*. *brunneum* 40 Δ** **Experiment**			
TE (s)	114.4±18.3	154.2±20.9	0.001
S (cm/s)	0.58±0.09	0.42±0.06	0.0001
SR (cm/s)	0.88±0.3	0.64±0.1	0.0009
***N*. *inversa* 60 Δ** **Control**			
TE (s)	93.2±37.6	85.7±32.6	0.4
S (cm/s)	0.82±0.3	0.86±0.3	0.72
SR (cm/s)	1.06±0.6	1.23±1	0.37
***N*. *inversa* 60 Δ** **Experiment**			
TE (s)	52.8±27.9	112.3±13.9	2.1e^-12^
S (cm/s)	1.64±1.05	0.58±0.07	1.0e^-8^
SR (cm/s)	1.89±0.9	0.96±0.1	5.8e^-8^

TE = time to get to the chosen end; S = mean speed of displacement inside the maze until reaching the resource; SR = speed of return to the nest; Δ = individuals; s = seconds; cm = centimetres. Values expressed as Mean ± Standard Deviation.

While ants of both species moved inside the maze when the coils were activated, they displayed body cleaning, antennal boxing and attempted seizure, body lifting and mandible opening (Table 4). In 75% and 85% of cases, workers of *N*. *inversa* and *E*. *brunneum*, respectively, stopped their movement and remained completely still inside the Y-maze remaining for a mean time of 15.71s ± 4.07s and 20.25s ± 8.48s, respectively, when under the influence of the applied MF.

**Table 4:** Frequencies of behaviours performed during interactions between workers in the decision-making experiments of two ant species with and without the effect of the applied MF.

Behaviours	Frequency during encounters (%)			
	*Ectatomma brunneum*		*Neoponera inversa*	
	NMF	AMF	NMF	AMF
Cleaning antennas	100	100	100	100
Cleaning legs	55	100	65	96.6
Cleaning abdomen	n/f	n/f	1.6	80
Body lifting	0	12.5	0	33.3
Antennal boxing	7.5	52.5	40	86.6
Attempted seizure	0	27.5	8.3	25
Opening mandible	10	0	18.3	10

NMF = Normal magnetic field; AMF = Applied magnetic field; n/f = not found.

**Fig 4:**
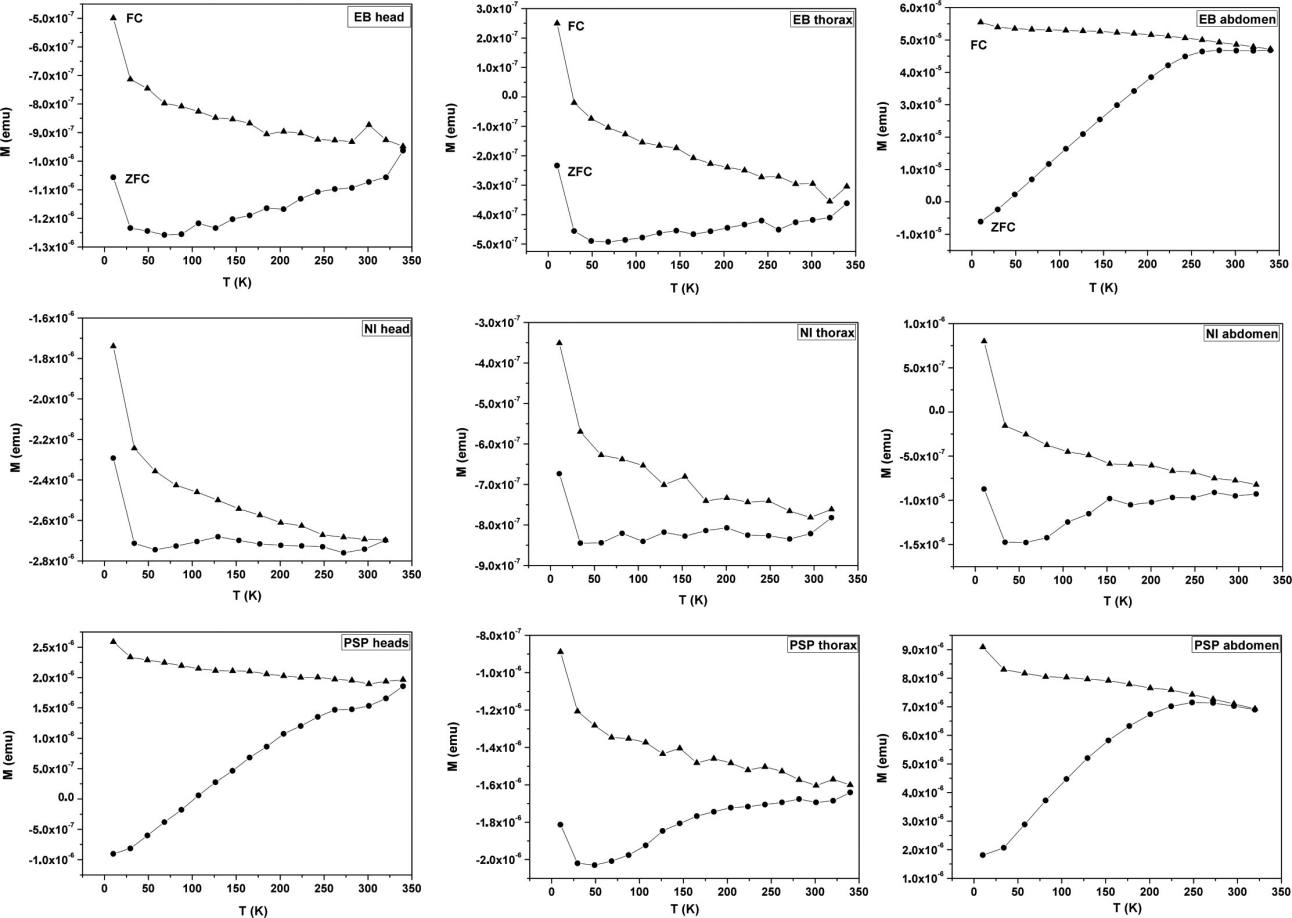
ZFC and FC curves from 10 K to 330 K in the presence of a magnetic field of 100 Oe. First column corresponds to head samples, second column to thorax samples and third column to abdomen samples. Graphs show the magnetization as function of temperature. An indication of the presence of magnetic nanoparticles in the body parts is that both curves are different. As a rule, ZFC curves are below FC curves because of the presence of a magnetic field during the cooling process to obtain the FC curve.

Fig 4 shows the results for the ZFC and FC curves obtained for all body parts of the ants PSP, EB and NI. Those curves show for the three body parts a behaviour compatible with the presence of magnetic nanoparticles; however, the magnetic behaviour of those nanoparticles is not the same among the parts. In EB the head and thorax curves are similar and different from those of the abdomen. In NI the head, thorax and abdomen curves are similar, but the ZFC curves of head and abdomen show a small transition around 125K. As explained before, this magnetic transition is a fingerprint for magnetite. In PSP the three body parts show different curves. In some cases, the magnetization is negative because the diamagnetism is stronger than the ferromagnetic component. From these curves, we can state that there are magnetic nanoparticles in the ants’ body; however, it is not possible to establish the value of the blocking temperature T_B_, because it is not possible to observe the irreversibility temperature T_irr_ as defined by Hansen and Morup in [51]. Apparently, the blocking temperature is similar to or greater than 300K. In the ZFC/FC curves, an increase in magnetization at low temperature is noted. This has been observed in nanoparticles of NiO and CuO and identified as a paramagnetic contribution due to spins not compensated on the surface of the nanoparticle [52]. That means that the magnetization measurement has diamagnetic, paramagnetic, superparamagnetic and stable ferromagnetic contributions. For a biological magnetosensor only superparamagnetic and stable ferromagnetic nanoparticles are interesting. The difference between both types is the size, because stable ferromagnetic nanoparticles are bigger than superparamagnetic nanoparticles, and because depending on the size the blocking temperature can be higher or lower than the ambient temperature. As T_B_ is similar or higher than 300 K, the superparamagnetic nanoparticles must be in the ferromagnetic state with a stable magnetic moment direction.

**Fig 5:**
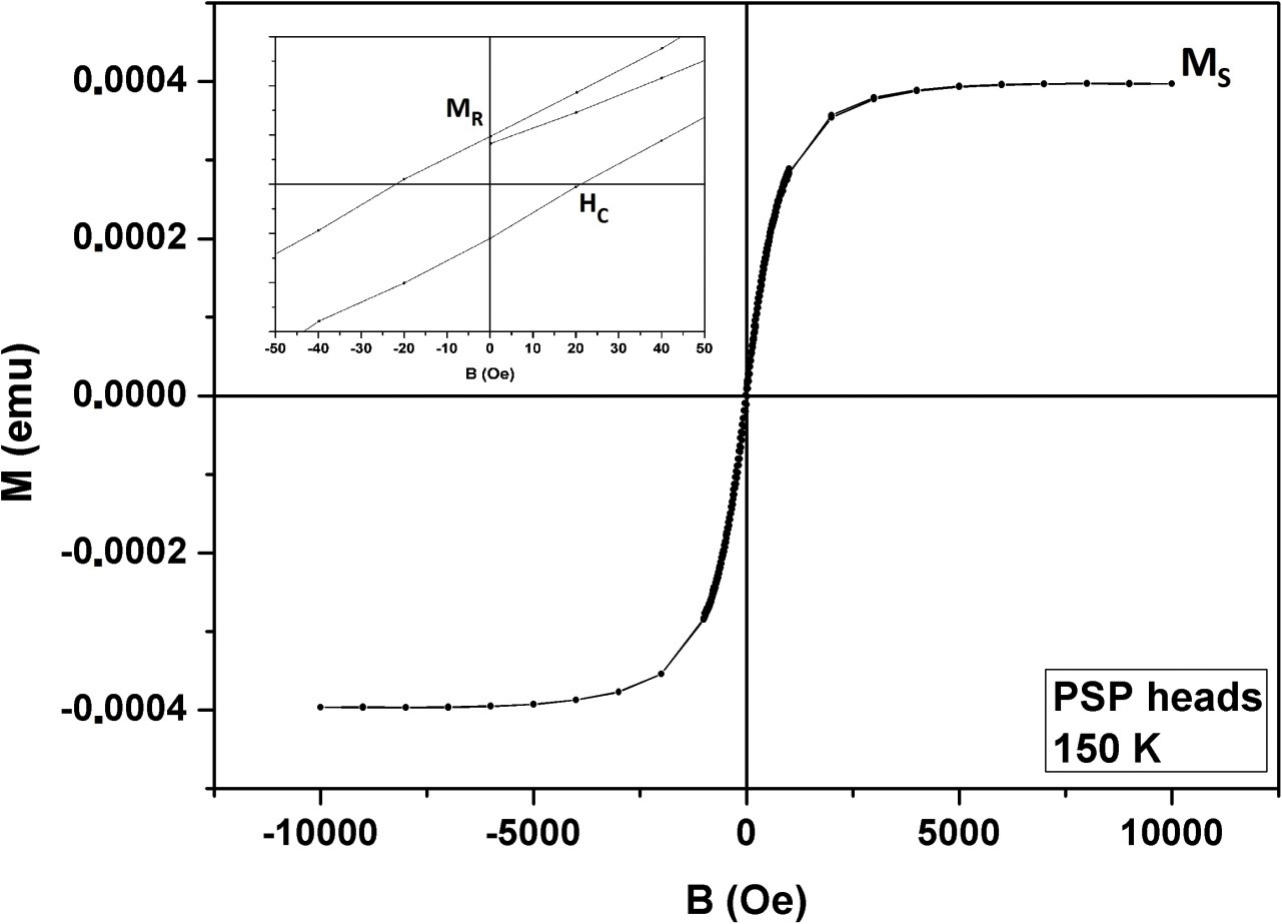
Example of hysteresis curve obtained for 20 heads of PSP ants at 150 K. The graph shows the magnetization M as a function of the magnetic field B. The magnetic field increases from zero to 10000 Oe. The insert shows an amplification in the region from -50 Oe to +50 Oe where is possible to observe the hysteresis behaviour related to the presence of ferromagnetic or blocked superparamagnetic nanoparticles. M_S_ = saturation magnetization; M_R_ = remanent magnetization; H_C_ = coercive magnetic field.

**Fig 6:**
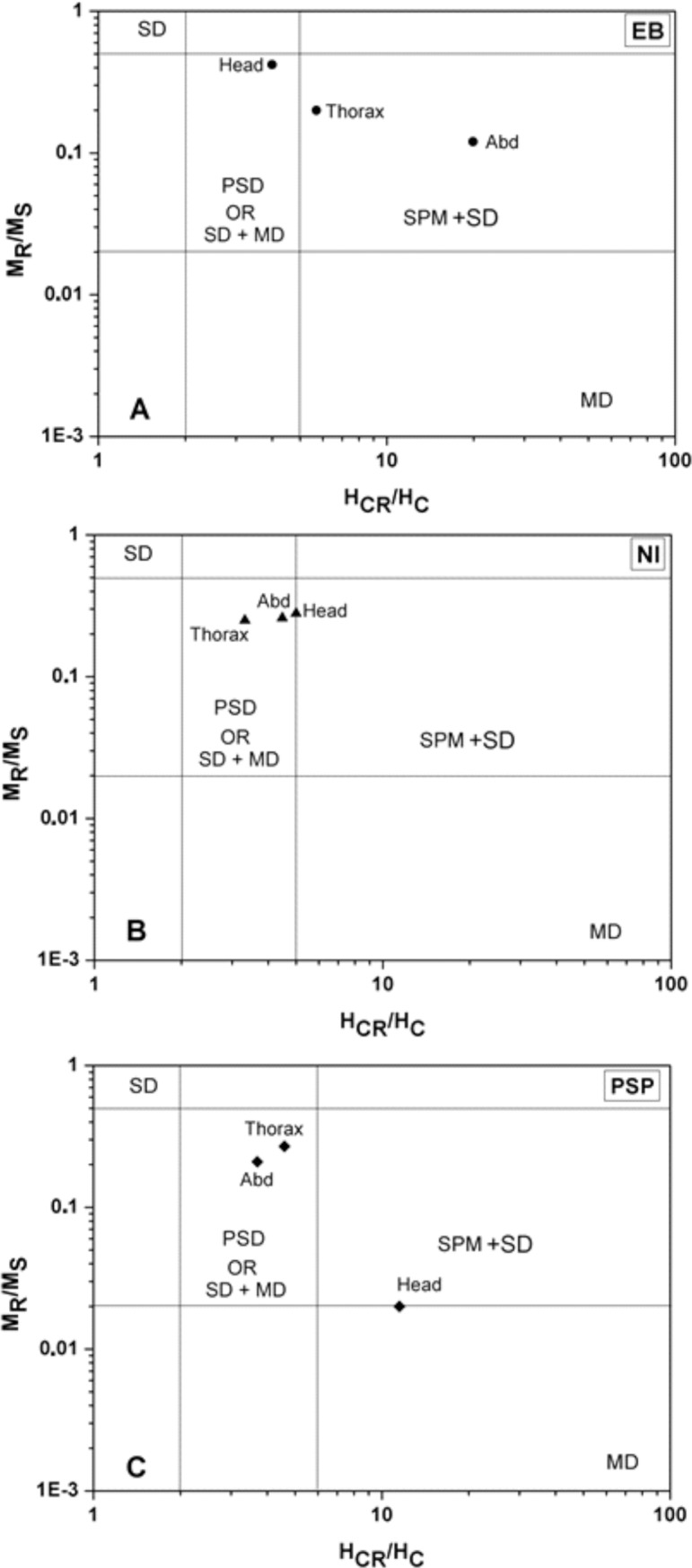
Day plot (M_R_/M_S_ vs H_CR_/H_C_) for the hysteresis data obtained for ant's parts. EB = *Ectatomma brunneum*; NI = *Neoponera inversa*; PSP = *Pheidole* sp.. The plot permits the identification of the type of nanoparticles that produced the hysteresis curve, dividing the plane in regions associated to magnetic domain types: SD = single domain; PSD = pseudo-single domain; MD = multidomain; SPM = superparamagnetic. The hysteresis curve for EB was obtained at 300 K and for NI and PSP at 150 K.

Once the existence of magnetic nanoparticles in the ant’s body was confirmed, hysteresis curves and the Day plot were done to obtain more information about the type of magnetic nanoparticles in the body parts. Fig 5 shows an example of a curve of magnetization M versus magnetic field B showing hysteresis, obtained for PSP heads at 150K after taking out the diamagnetic contribution. In this figure we can observe the saturation magnetization (M_S_), the remanent magnetization (M_R_) and the coercive field (H_C_). The presence of M_R_ and H_C_ in these curves is characteristic of ferromagnetic material or blocked superparamagnetic nanoparticles. Table 5 shows the values of M_S_ and M_R_ for the different body parts of PSP, EB and NI. As the parts are very different in size, values relative to the mass (M_S,R REL_) were obtained by the ratio M_S,R_/mass for each body part. In all cases, the abdomen had the highest values of M_R REL_, meaning that in this body part is found the greatest relative amount of ferromagnetic nanoparticles. M_S REL_ is greater in the abdomen of EB and NI and in the head of PSP, indicating that it is in these body parts that most of the superparamagnetic, ferromagnetic and paramagnetic nanoparticles are found. As only superparamagnetic and ferromagnetic nanoparticles have been considered to be part of a magnetosensor it is interesting to classify the type of nanoparticle by the parameters of the hysteresis curve. To do that it was used the Day plot as was explained before. Fig 6 shows the Day plot built for the different body parts of PSP, EB and NI ants using the data obtained for their respective hysteresis curves. In the three ant species, the magnetic nanoparticles are among a mixture of SD and SPM particles or SD and MD particles. In the ants PSP and EB the thorax and abdomen have similar types of magnetic nanoparticles while the head nanoparticles are in another region.

**Table 5:** Magnetization parameters obtained from the hysteresis curves performed for abdomens, thoraxes and heads.

Body part	N	Mass (g)	M_R_ (emu)	M_R REL_ (emu/g)	M_S_ (emu)	M_S REL_ (emu/g)
**EB (300K)**						
Abdomen	5	0.0134	2.32x10^-5^	0.0017	1.92x10^-4^	0.0143
Thorax	5	0.0158	3.97x10^-6^	0.00025	1.95x10^-5^	0.00122
Head	5	0.0094	4.5x10^-6^	0.00047	1.06x10^-6^	0.00112
**NI (150 K)**						
Abdomen	4	0.0073	4.9x10^-6^	0.00066	1.84x10^-5^	0.0025
Thorax	4	0.0065	3x10^-6^	0.00046	1.2x10^-5^	0.0018
Head	4	0.0079	2.3x10^-6^	0.00025	8x10^-6^	0.0010
**PSP (150K)**						
Abdomen	20	0.00072	7.5x10^-6^	0.01	3.5x10^-5^	0.048
Thorax	20	0.0028	3.8x10^-6^	0.0013	1.4x10^-5^	0.005
Head	20	0.0017	1x10^-5^	0.0059	3.9x10^-4^	0.229

N = number of parts used in the measurement; EB = *Ectatomma brunneum*; NI = *Neoponera inversa*; PSP = *Pheidole* sp.. The hysteresis curves were performed at 300 K or 150 K and is indicated next to the ant's name. The saturation magnetization (M_S_) and remanent magnetization (M_R_) are represented in Fig 5. The mass corresponds to the N parts used. M_R,S REL_ = (M_R,S_)/Mass.

## Discussion

According to our results, we can conclude that an applied MF affects the foraging behaviour and decision-making of worker ants, from ant species that have mass recruitment or forage individually.

The OVS results show that the applied MF affect the trajectory of W1 and W2 of *Pheidole* sp. (Fig 3). This is very interesting, because the W2 uses mainly chemical stimuli to guide its trajectory. The increase in the local magnetic field might create some kind of disturbance in the ant chemical sensors, which causes the modifications observed in the parameters of the trajectories.

The significant change in trajectory and behaviour of ants under the effect of the applied MF, changing significantly the trajectory, mean speed of displacement, detection time of resource and flow of ants, suggest that these ants are magnetosensitive. Anderson and Vander Meer [37] observed that the change of direction of the geomagnetic field can affect the time taken to form a trail in the search of food and return to the colonies in *S*. *invicta*.

The effects of the applied MF are only felt by the ants when they are close to the coils (Fig 3); however, persist at least until they return to the nest. The fact that the ants remain significantly less time in the resource when the coils are activated (Tables 1 and 2), suggests that the idea that the applied MF might generate some kind of disturbance in the ant body, as documented by Kermarrec [36] who assessed the effects of magnetic fields on *A*. *octospinosus* and observed that altered MF leads to changes in allocation of immature ants and workers in the colonies. However, unlike Kermarrec [36] the MF used in our study is small, because the increase in the horizontal component of the MF to 60 μT, together with the vertical component of -10.6 μT, cause an increase in the total magnetic field to 60.9 μT, which corresponds to approximately 3 times the normal geomagnetic field. At the same time, the inclination of the magnetic field changes from -28° to -10°.

On the other hand, we cannot rule out the fact that the ants could have changed their behaviour due to some kind of stress caused by the coils, as these devices can increase the temperature of the space between them and some animals feature temperature-bound behavioural changes [53–55]. However, despite the lack of studies on the effects of the heat generated by the coils, we ran tests that show that the body heat of ants under these conditions do not increase significantly compared to the condition when the coils are switched off, although these results are not part of this study.

The decrease in the flow of ants (Table 1) reinforces the fact that the applied MF might generate disturbances in ants and leave them unable to follow the chemical signals deposited on the trails (Fig 3). Indeed, the study by Banks and Srygley [41] shows that to some degree, leaf-cutting ants can change their behaviour of following chemical trails when affected by a magnetic field.

Another interesting behaviour that shows the magnetosensitivity in the studied ants, is that when they approach the resource in the area between the activated coils, they stop and remain completely immobile for about twenty seconds. A similar disturbance is also observed during the decision-making tests when the ants chose most of the times the Y arm with the resource and normal MF (Table 3). The time and the speed of return to the nest are significantly different when the coils are activated. It seems, therefore, that by the time the ants perceive the applied MF they tend to return as soon as possible, suggesting once again some type of disturbance and they also perform atypical behaviours to those displayed in their normal foraging repertoire [56,57].

The time spent searching for food resources are significantly different between the conditions with coils off and with coils activated. This can be explained, because once ants spent more time in the arm of Y-maze under the influence of the applied MF, maybe because the applied MF might have caused some kind of disorientation or disturbance in the ant that took more time to evaluate the path, capture the resource and return to the nest.

All these results indicate that the three species of ants tested here are magnetosensitive. The particles detected in the different body parts of the ants support this hypothesis. The curves found in the magnetometry measurements are characteristic of magnetic nanoparticles [51,58] and indicate the presence of these particles in the whole body of the ants. One of the models accepted for the detection of magnetic fields by animals is the ferromagnetic hypothesis, which states that the magnetic field is transduced by magnetic nanoparticles attached to cells in contact with the nervous system [2]. Recent studies have shown that in the ants *N*. *marginata* the magnetosensor may be located in the antennae [59] and not necessarily have to be composed by biomineralized magnetite [9]. In our study, the presence of magnetite is observed only in *N*. *inversa* by the Verwey transition (Fig 4) while in the other species this transition is not observed. In this species, the ZFC curves of head and abdomen show a small transition around 125K that can be related to the Verwey transition, around 120K, of the magnetic iron oxide magnetite [58]. As is not expected the magnetosensor to be in the thorax, it is possible that the presence of magnetic material is due to soil contamination.

The abdomen and thorax of PSP and EB have the same type of behaviour in the Day plot, which may imply that both share the same type of nanoparticles, and the head is located in a region with different magnetic properties. In the case of NI, that distinction among all parts is not very clear. In all ants analysed, the abdomen shows the highest values of M_R REL_ implying that it has the highest proportion of ferromagnetic nanoparticles, and perhaps corresponds to ingested material. In PSP, the head has the greatest value of M_S REL_ implying that there are extra contributions of particles in superparamagnetic state in addition to the ferromagnetic. For EB and PSP we can assume that the magnetic sensor should be located in the head, because their nanoparticles are distinguished from abdomen and thorax in the Day plot, with the heads of EB presenting mixtures of SD and MD nanoparticles or PSD nanoparticles; and the heads of PSP having mixtures of SD and SPM nanoparticles. This assumption is based also in the proposal done for *N*. *marginata* where also is observed magnetic material in all the body but the antenna shows higher values of M_S REL_, being distinguished from the other body parts [59,60]. In the ants studied in the present research only for EB and PSP ants the head nanoparticles are different from the others present in thorax and abdomen. For NI ants the abdomen shows the higher values of M_S REL_ and M_R REL_, perhaps being the magnetosensor location.

Interestingly, in the ZFC and FC plots for all thoraxes the magnetization increases at low temperatures. That can be associated with a paramagnetic contribution created by surface defects [52], which may be indicative of contamination by soil nanoparticles. The same plots show that in NI the three body parts show this increase in magnetization at low temperature and also show the characteristic transition of magnetite, which may imply that the whole body of these ants was contaminated with nanoparticles of magnetite, which does not exclude that in the NI abdomen or head there is a magnetization contribution from nanoparticles associated with a magnetic sensor. In addition, the presence of magnetic nanoparticles can be associated to other functions, not only to magnetoreception, as for example, cuticle hardening as occurs in chiton teeth [61]. Our magnetometry measurements do not discard the presence of a magnetic sensor, based on magnetic nanoparticles, in the head or abdomen.

All the ants analysed in this study showed sensitivity to the applied magnetic field. This change represented an increase of intensity of 3 times the geomagnetic field and a change in the inclination from -28° to -10°.

The change in the parameters of the trajectories observed in the ants W2, the first worker recruited to follow the chemical trail, under the applied magnetic field might mean that W2 uses chemical and magnetic detection to guide its trajectories. Also, our results show that the change in the magnetic field altered behaviours associated with spatial orientation and detection of chemical markers. Apparently, ants have a magnetic detection system calibrated to the parameters of the local geomagnetic field, because the increase of intensity in three times the local MF produced a low intensity MF that can be found normally in the geomagnetic field of Northern Canada, Northern Russia, and South Australia, for example in Melbourne and Tasmania island. The new inclination of -10° is typical of Northern South America and central Africa. Models for detection with magnetic nanoparticles consider the presence of SD or SPM particles [3]. As the magnetic field increase is low, the more likely is that SPM nanoparticles are associated with the magnetic sensor. However, if the effects observed are associated with the change in inclination, then the inclination compass that functions through the mechanism of radical pairs can be affected in the antenna [6]. It is difficult to establish a link between the chemical and magnetic detection mechanism in the antenna, because little is known of both, but this type of relationship is not impossible since it happens in magnetotactic bacteria in the behaviour known as magneto-aerotaxis, where the magnetotaxis works along with oxygen detection [62].

## Conclusions

The present study shows that the three ant species are magnetosensitive, and were affected by changes of low intensity in the local magnetic field. For *Pheidole* sp. the magnetic field affects the navigation performance, and in consequence magnetoreceptive behaviour. Also, our experiments show that *Pheidole* sp., *E*. *brunneum* and *N*. *inversa* ants changes their foraging strategies when the magnetic field changed. The presence of magnetic nanoparticles in the different body parts suggest that the magnetic field transduction can be done through the ferromagnetic hypothesis mechanism. However, the presence of magnetic nanoparticles in the ant's body does not discard the use of other magnetoreception mechanisms by the ants, such as the radical pair mechanism. As there are few studies on the effects of applied MF of low intensity on the behaviour of social insects, we cannot rule out the possibility that they are probably the product of some kind of stress generated by the devices used to change the intensity of the magnetic field, although previous tests show that they’re probably not. Thus, new studies are required to clarify the physical detection mechanisms and their impact on the maintenance of the colonies.

## Supporting information

S1 Fig Trajectories of two *Pheidole* sp. workers foraging under normal (NMF) and applied (AMF) magnetic field. (A) Patterns of departure and return to the nest trajectories for Nest 1 under normal MF (B) Patterns of departure and return to the nest trajectories for Nest 1 under applied MF.
(TIF)

S2 Fig Trajectories of two *Pheidole* sp. workers foraging under normal (NMF) and applied (AMF) magnetic field. (A) Patterns of departure and return to the nest trajectories for Nest 2 under normal MF (B) Patterns of departure and return to the nest trajectories for Nest 2 under applied MF.
(TIF)

S3 Fig Trajectories of two *Pheidole* sp. workers foraging under normal (NMF) and applied (AMF) magnetic field. (A) Patterns of departure and return to the nest trajectories for Nest 3 under normal MF (B) Patterns of departure and return to the nest trajectories for Nest 3 under applied MF.
(TIF)

S4 Fig Trajectories of two *Pheidole* sp. workers foraging under normal (NMF) and applied (AMF) magnetic field. (A) Patterns of departure and return to the nest trajectories for Nest 4 under normal MF (B) Patterns of departure and return to the nest trajectories for Nest 4 under applied MF.
(TIF)

S5 Fig Trajectories of two *Pheidole* sp. workers foraging under normal (NMF) and applied (AMF) magnetic field. (A) Patterns of departure and return to the nest trajectories for Nest 5 under normal MF (B) Patterns of departure and return to the nest trajectories for Nest 5 under applied MF.
(TIF)

S6 Fig Trajectories of two *Pheidole* sp. workers foraging under normal (NMF) and applied (AMF) magnetic field. (A) Patterns of departure and return to the nest trajectories for Nest 6 under normal MF (B) Patterns of departure and return to the nest trajectories for Nest 6 under applied MF.
(TIF)

S7 Fig Trajectories of two *Pheidole* sp. workers foraging under normal (NMF) and applied (AMF) magnetic field. (A) Patterns of departure and return to the nest trajectories for Nest 7 under normal MF (B) Patterns of departure and return to the nest trajectories for Nest 7 under applied MF.
(TIF)

S8 Fig Trajectories of two *Pheidole* sp. workers foraging under normal (NMF) and applied (AMF) magnetic field. (A) Patterns of departure and return to the nest trajectories for Nest 8 under normal MF (B) Patterns of departure and return to the nest trajectories for Nest 8 under applied MF.
(TIF)

S9 Fig Trajectories of two *Pheidole* sp. workers foraging under normal (NMF) and applied (AMF) magnetic field. (A) Patterns of departure and return to the nest trajectories for Nest 9 under normal MF (B) Patterns of departure and return to the nest trajectories for Nest 9 under applied MF.
(TIF)

S10 Fig Trajectories of two *Pheidole* sp. workers foraging under normal (NMF) and applied (AMF) magnetic field. (A) Patterns of departure and return to the nest trajectories for Nest 10 under normal MF (B) Patterns of departure and return to the nest trajectories for Nest 10 under applied MF.
(TIF)
