# Supplementary figures and images for: Can altered magnetic field affect the foraging behaviour of ants?
Source: PLoS One. 2019 Nov 25;14(11):e0225507. doi: 10.1371/journal.pone.0225507 (PMC6876837; doi:10.1371/journal.pone.0225507)

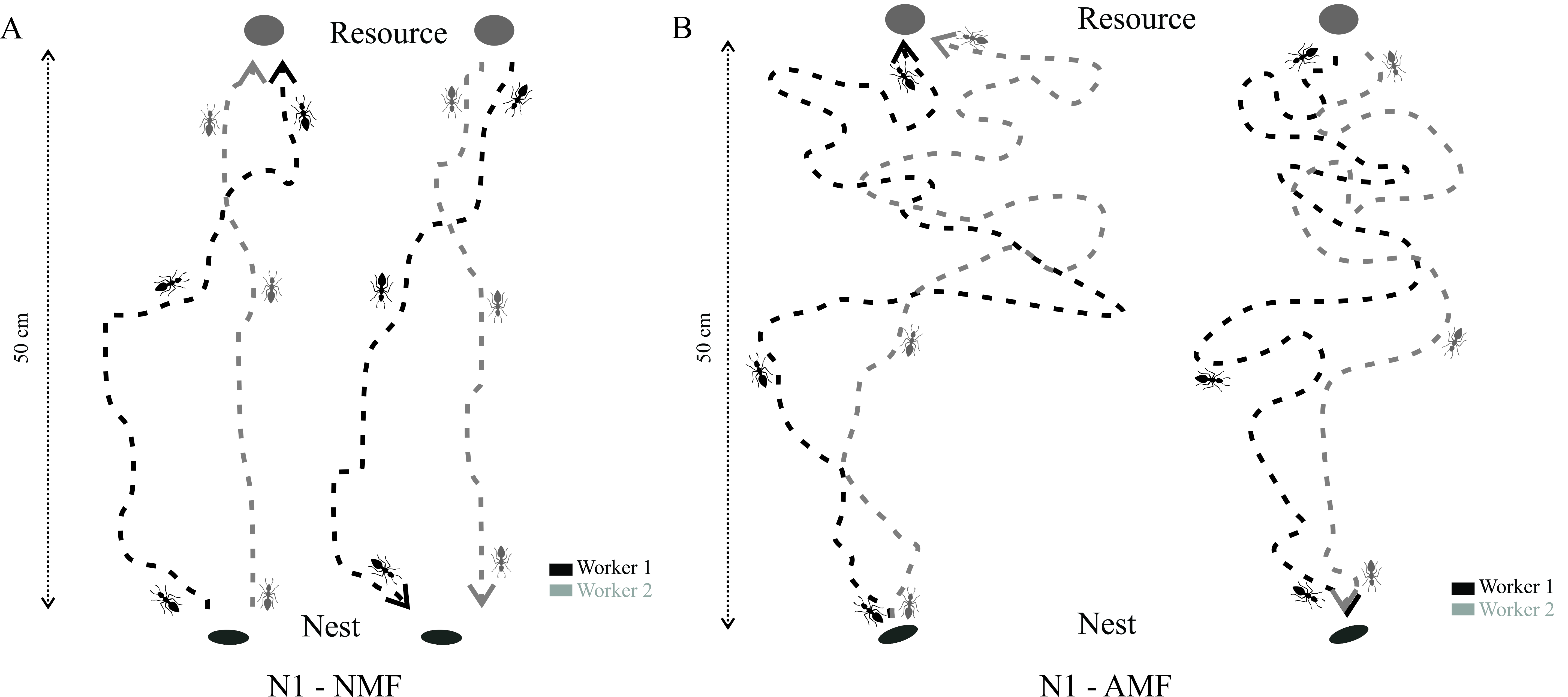

Supplement: S1 Fig — (A) Patterns of departure and return to the nest trajectories for Nest 1 under normal MF (B) Patterns of departure and return to the nest trajectories for Nest 1 under applied MF. (TIF) [file pone.0225507.s001.tif]

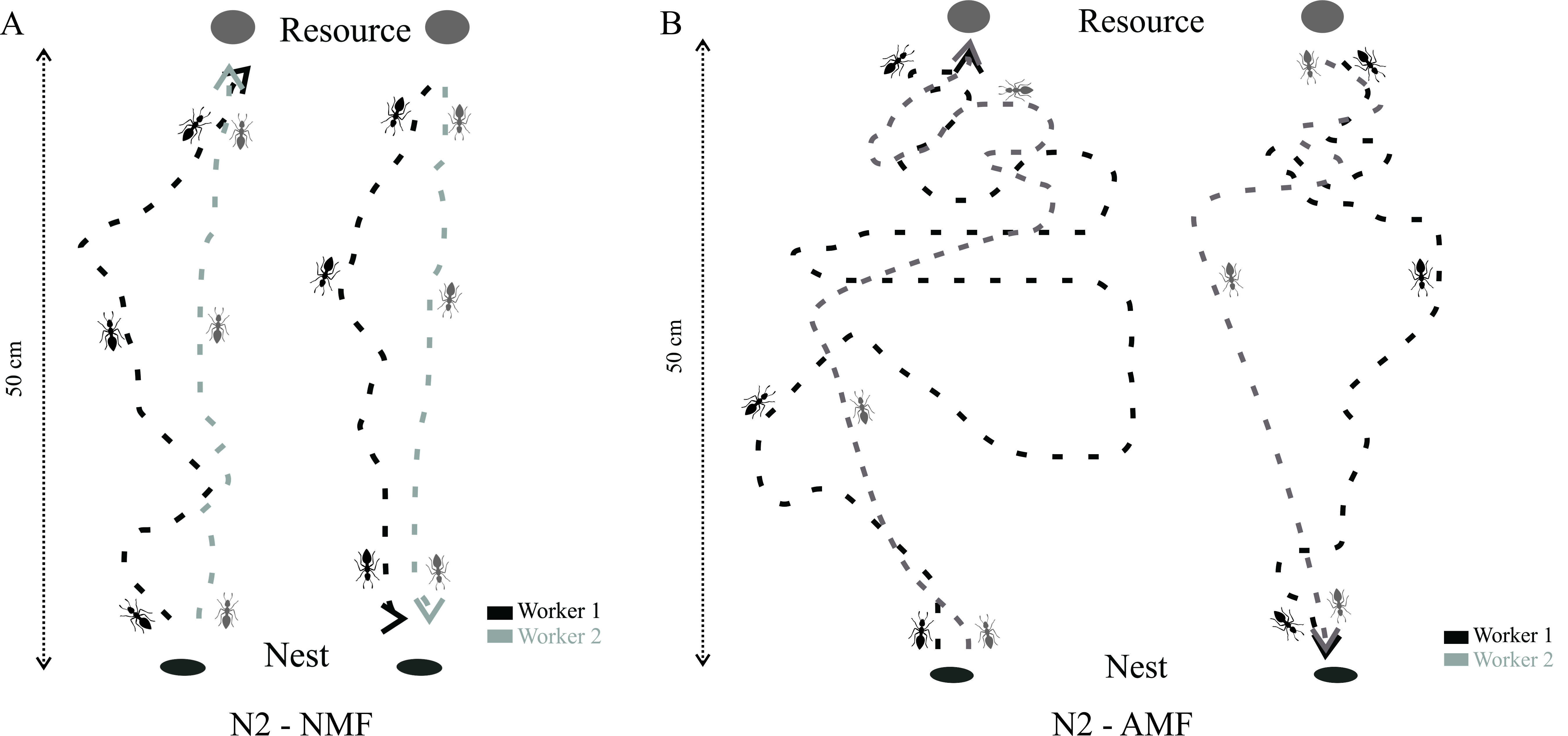

Supplement: S2 Fig — (A) Patterns of departure and return to the nest trajectories for Nest 2 under normal MF (B) Patterns of departure and return to the nest trajectories for Nest 2 under applied MF. (TIF) [file pone.0225507.s002.tif]

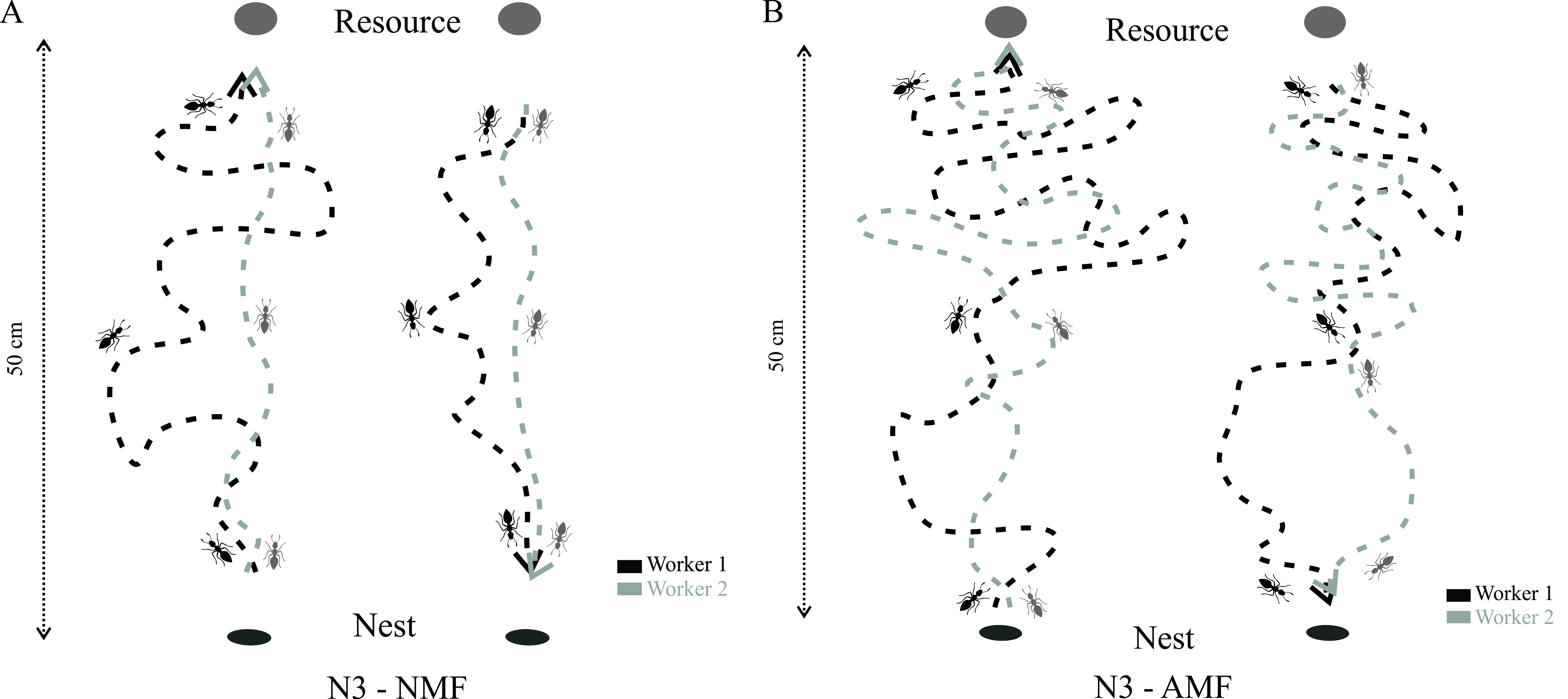

Supplement: S3 Fig — (A) Patterns of departure and return to the nest trajectories for Nest 3 under normal MF (B) Patterns of departure and return to the nest trajectories for Nest 3 under applied MF. (TIF) [file pone.0225507.s003.tif]

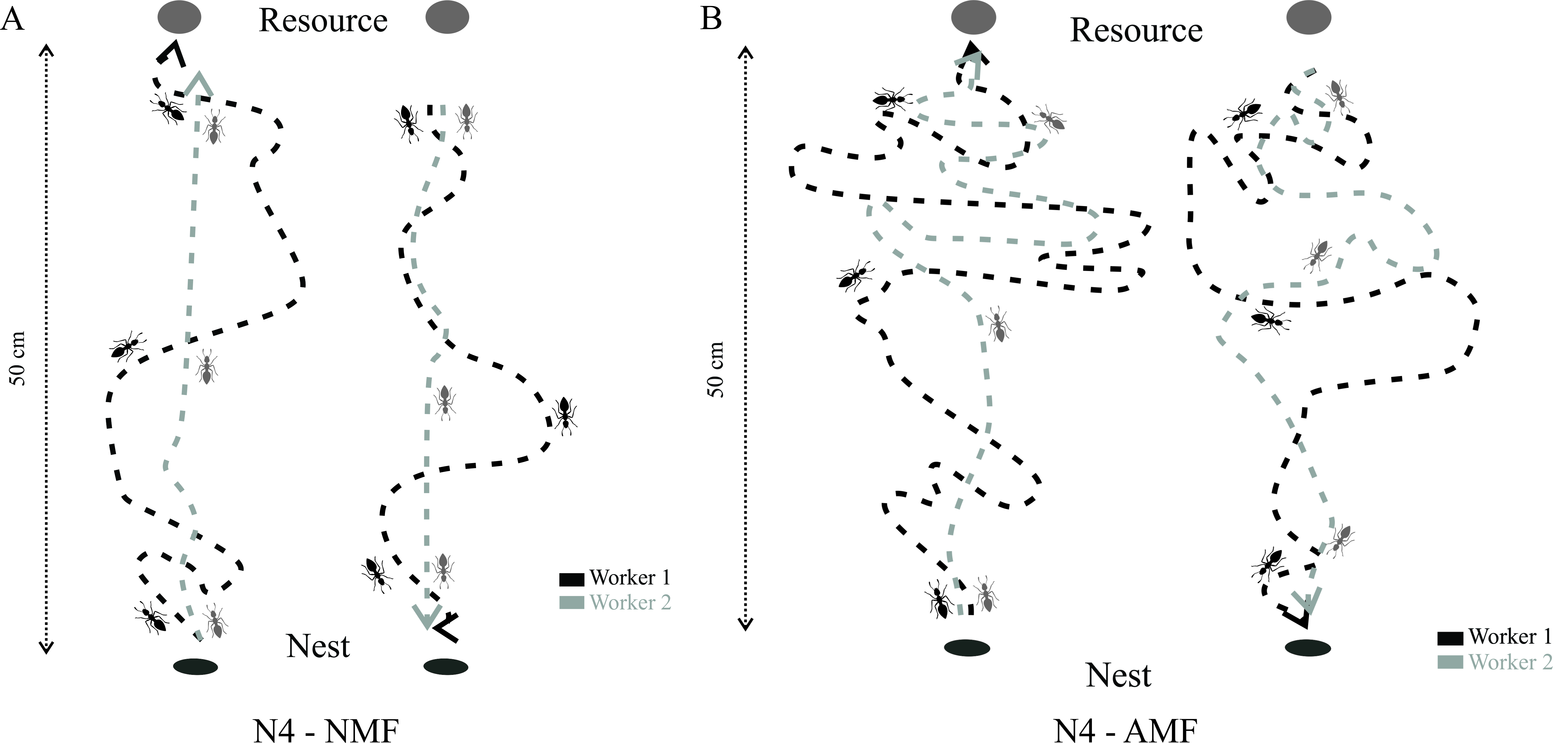

Supplement: S4 Fig — (A) Patterns of departure and return to the nest trajectories for Nest 4 under normal MF (B) Patterns of departure and return to the nest trajectories for Nest 4 under applied MF. (TIF) [file pone.0225507.s004.tif]

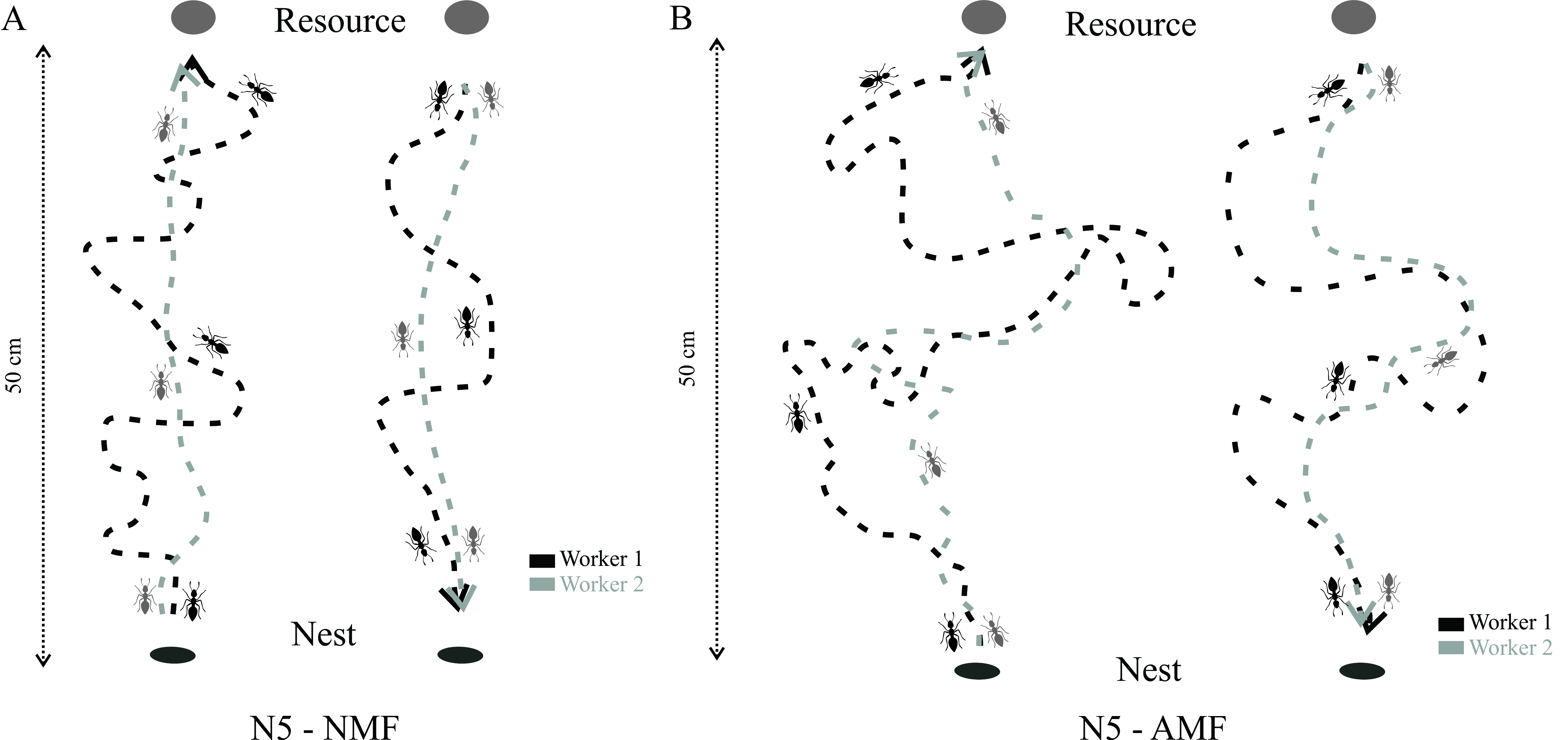

Supplement: S5 Fig — (A) Patterns of departure and return to the nest trajectories for Nest 5 under normal MF (B) Patterns of departure and return to the nest trajectories for Nest 5 under applied MF. (TIF) [file pone.0225507.s005.tif]

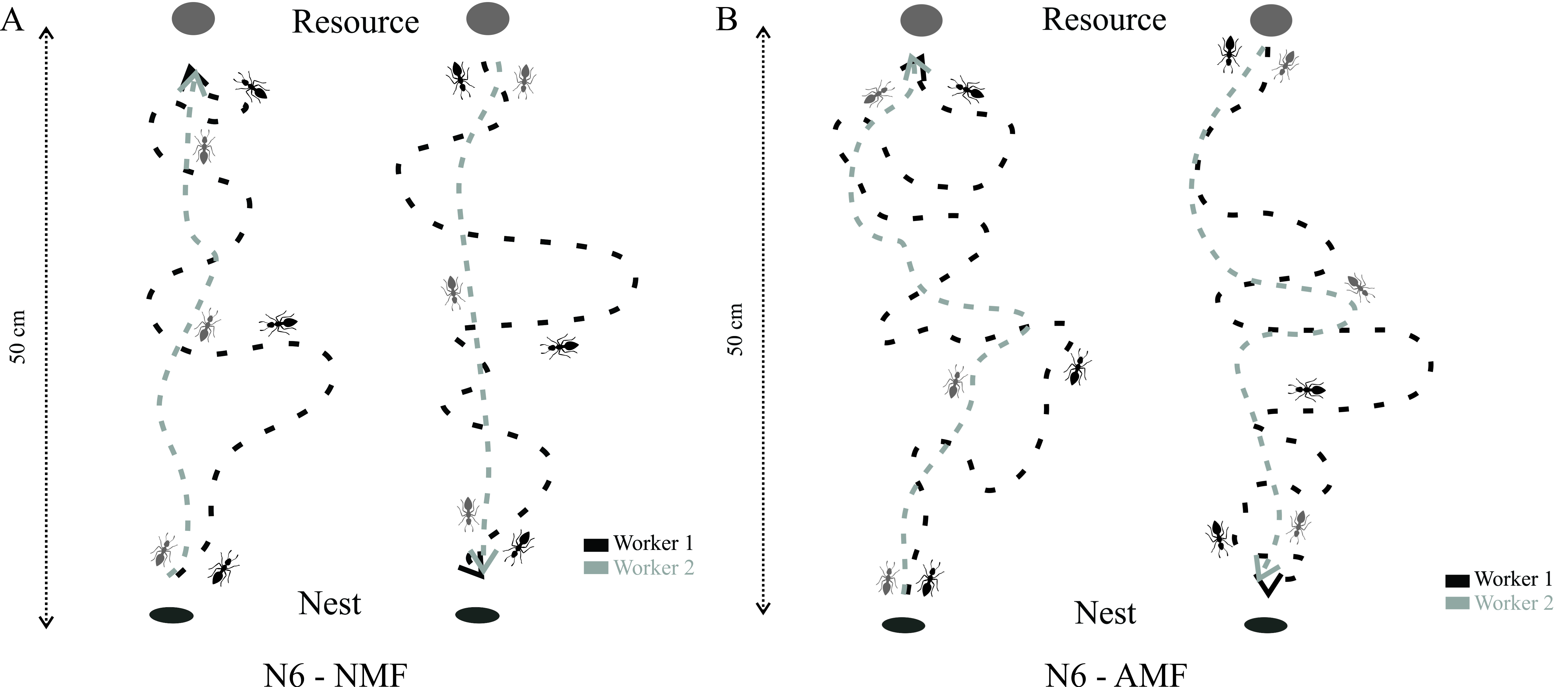

Supplement: S6 Fig — (A) Patterns of departure and return to the nest trajectories for Nest 6 under normal MF (B) Patterns of departure and return to the nest trajectories for Nest 6 under applied MF. (TIF) [file pone.0225507.s006.tif]

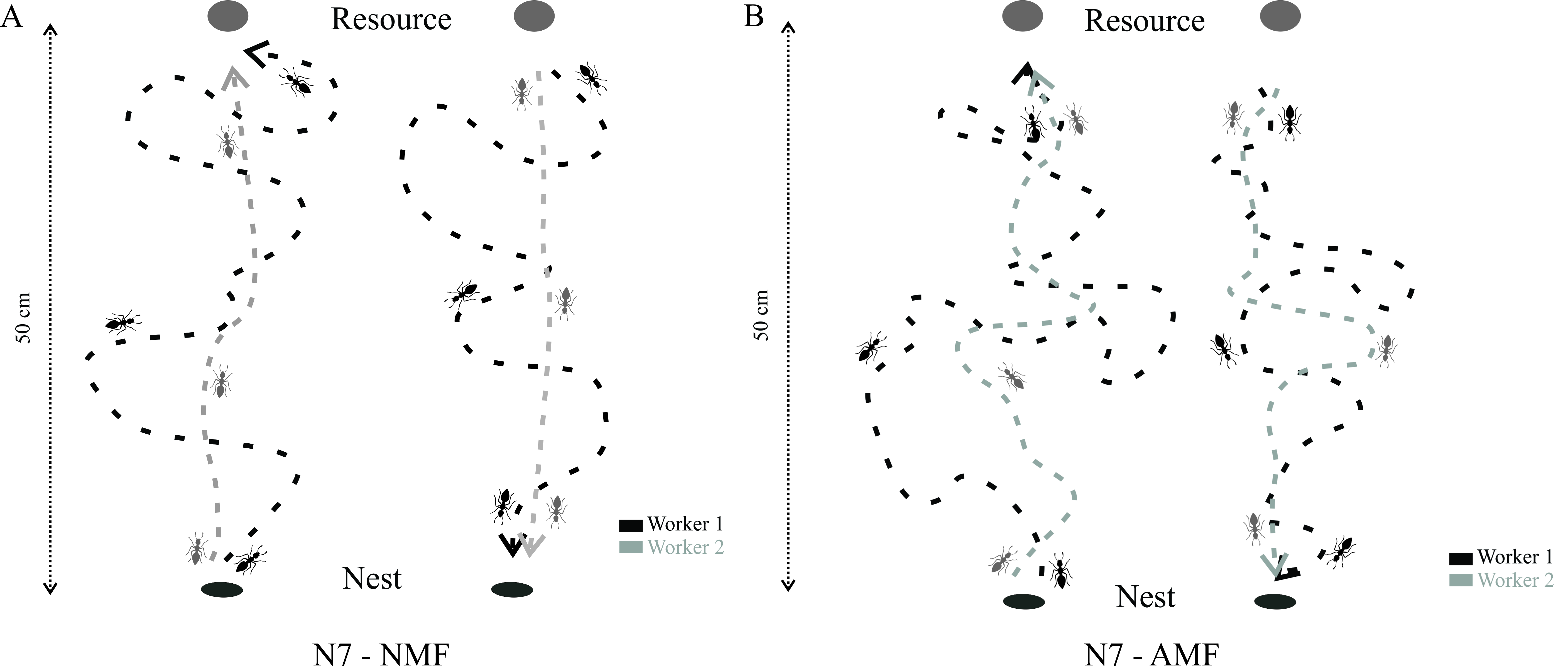

Supplement: S7 Fig — (A) Patterns of departure and return to the nest trajectories for Nest 7 under normal MF (B) Patterns of departure and return to the nest trajectories for Nest 7 under applied MF. (TIF) [file pone.0225507.s007.tif]

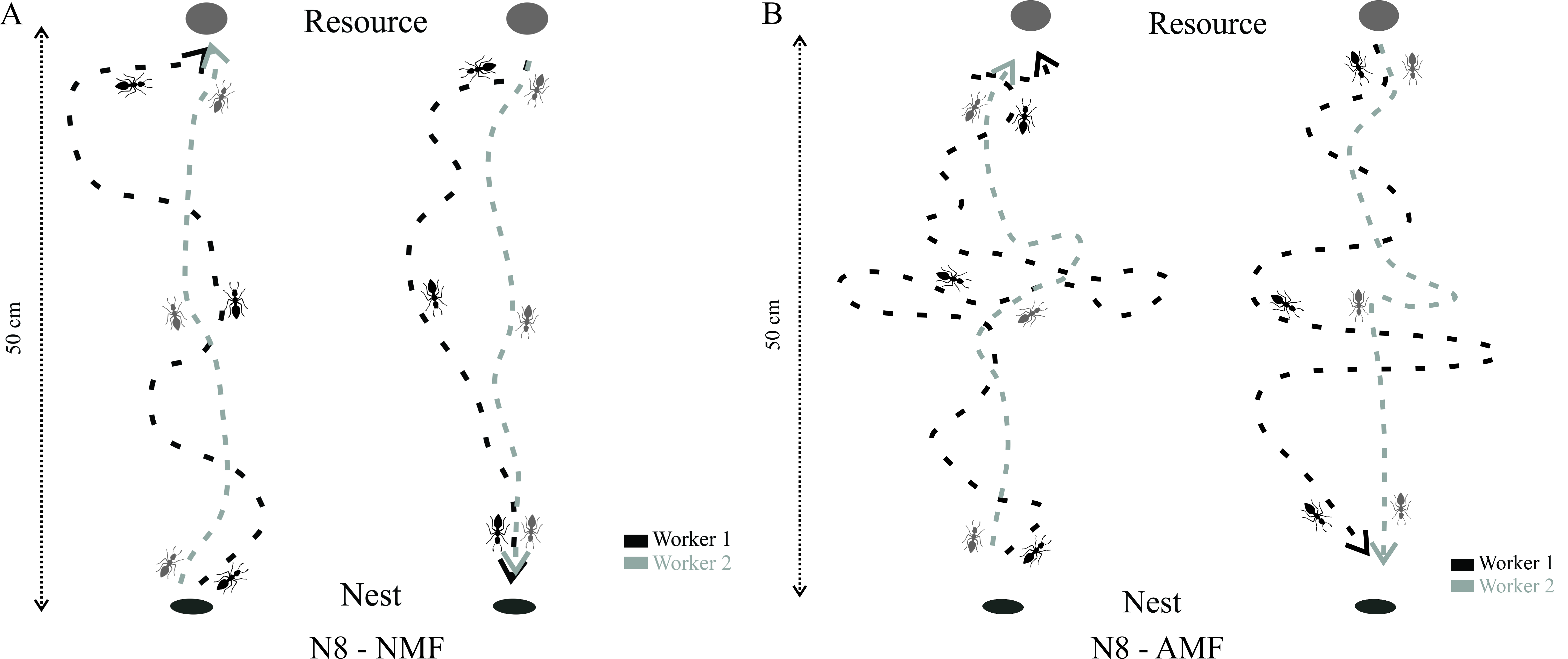

Supplement: S8 Fig — (A) Patterns of departure and return to the nest trajectories for Nest 8 under normal MF (B) Patterns of departure and return to the nest trajectories for Nest 8 under applied MF. (TIF) [file pone.0225507.s008.tif]

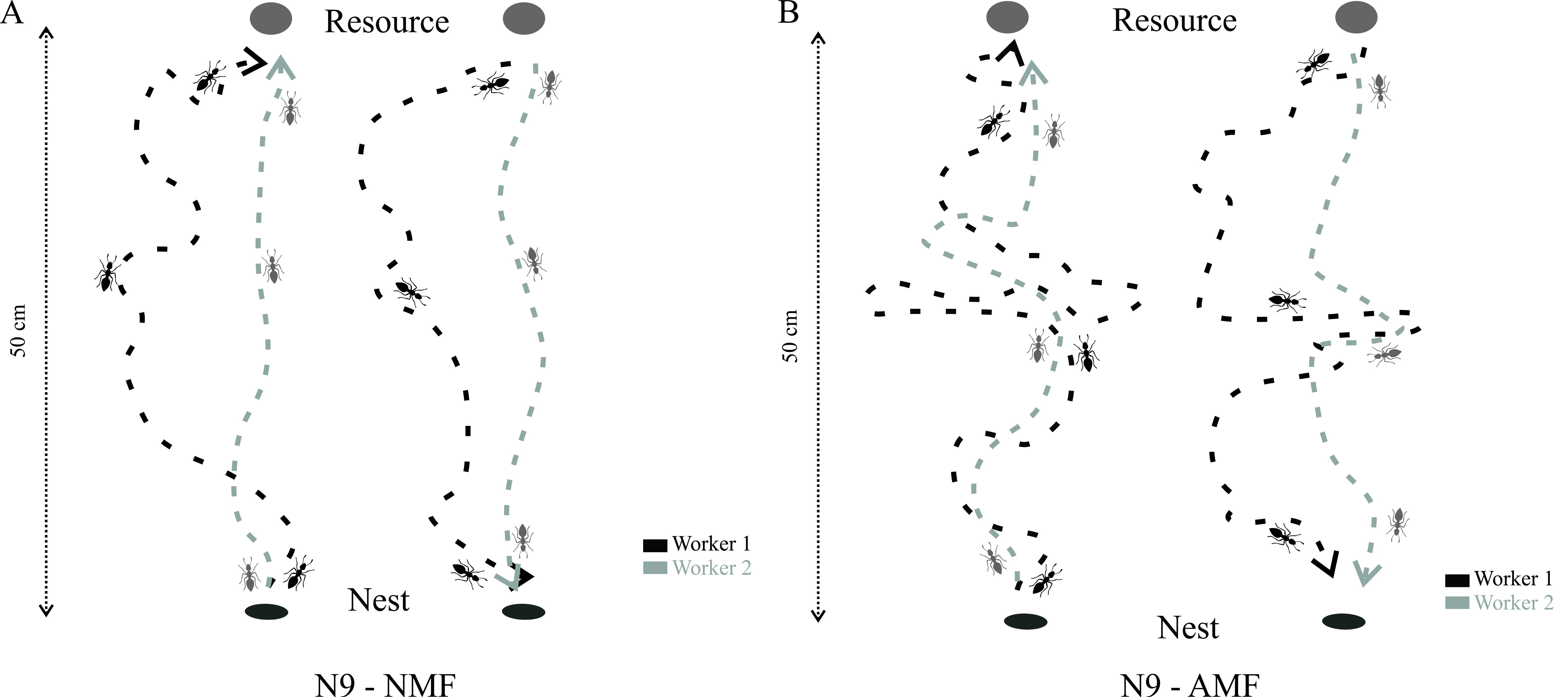

Supplement: S9 Fig — (A) Patterns of departure and return to the nest trajectories for Nest 9 under normal MF (B) Patterns of departure and return to the nest trajectories for Nest 9 under applied MF. (TIF) [file pone.0225507.s009.tif]

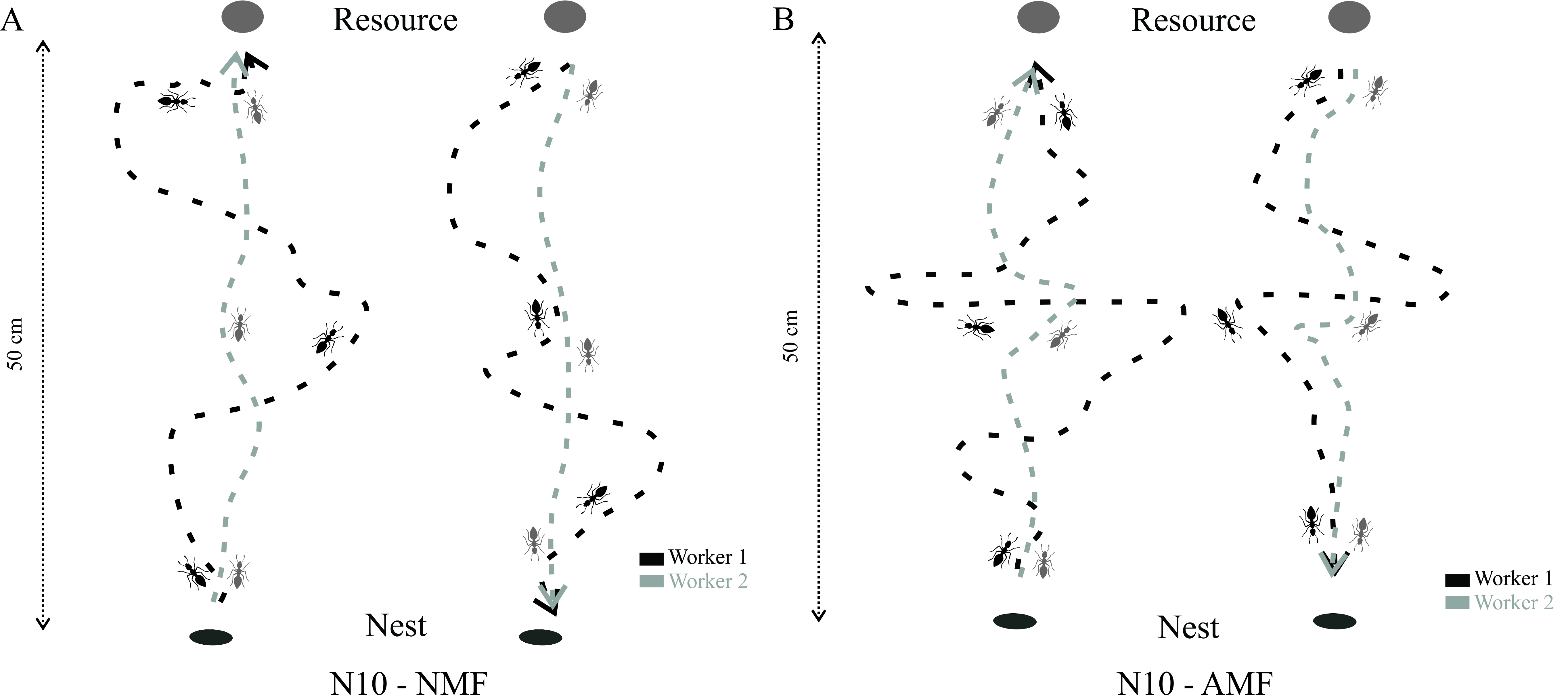

Supplement: S10 Fig — (A) Patterns of departure and return to the nest trajectories for Nest 10 under normal MF (B) Patterns of departure and return to the nest trajectories for Nest 10 under applied MF. (TIF) [file pone.0225507.s010.tif]
